# Quantification of Antioxidant Phenolic Compounds in a New Chrysanthemum Cultivar by High-Performance Liquid Chromatography with Diode Array Detection and Electrospray Ionization Mass Spectrometry

**DOI:** 10.1155/2017/1254721

**Published:** 2017-05-24

**Authors:** Ah-Reum Han, Hyo Young Kim, Yangkang So, Bomi Nam, Ik-Soo Lee, Joo-Won Nam, Yeong Deuk Jo, Sang Hoon Kim, Jin-Baek Kim, Si-Yong Kang, Chang Hyun Jin

**Affiliations:** ^1^Advanced Radiation Technology Institute, Korea Atomic Energy Research Institute, Jeongeup-si, Jeollabuk-do 56212, Republic of Korea; ^2^College of Pharmacy, Chonnam National University, Gwangju 11686, Republic of Korea; ^3^College of Pharmacy, Yeungnam University, Gyeongsan-si, Gyeongsangbuk-do 38541, Republic of Korea

## Abstract

The flowers of* Chrysanthemum morifolium* Ramat. have been used as an herbal tea and in traditional medicine, and the plant has been developed to produce horticultural cultivars of various colors and shapes. In this study, a new chrysanthemum cultivar with dark purple petals (*C. morifolium* cv. ARTI-Dark Chocolate; ADC) was developed by radiation-induced mutation breeding of its original cultivar with purple striped white petals (*C. morifolium* cv. Noble Wine, NW). The phenolic profile and antioxidant property of ADC were investigated and compared with NW and the commercially available medicinal herb,* C. morifolium* with yellow petals (CM), in order to find a scientific support to produce a new source of natural antioxidant. Flavonoid and phenolic acid profiles of the ethanol extracts of the three flowers were analyzed by high-performance liquid chromatography-diode array detector-electrospray ionization mass spectrometry (HPLC-DAD-ESIMS), while antioxidant properties were evaluated using the 1,1-diphenyl-2-picryl-hydrazyl (DPPH) and 2,2-azino-bis-3-ethylbenzothiazoline-6-sulfonic acid (ABTS) radical scavenging assay. Among the tested flowers, ADC possessed the strongest antioxidant capacity and the highest phenolic contents. Flavonoids (acacetin, apigenin, luteolin, acacetin-7-*O*-*β*-glucoside, apigenin-7-*O*-*β*-glucoside, luteolin-7-*O*-*β*-glucoside, and linarin) and phenolic acids (chlorogenic acid and mixture of 1,4-, 1,5-, and 3,5-dicaffeoylquinic acids) were identified and quantified.

## 1. Introduction

The dried flowers of* Chrysanthemum morifolium* Ramat. (Compositae), known as Chrysanthemi Flos, have been used as an infusion tea and in traditional medicine to treat inflammation, arteriosclerosis, and hypertension [1]. In previous phytochemical studies on* C*.* morifolium*, flavonoids and phenolic acids have been identified as major constituents [2], which have exhibited diverse biological activities such as antioxidant [3], anti-inflammatory [4], and antitumor effects [5].

**Figure 1:**
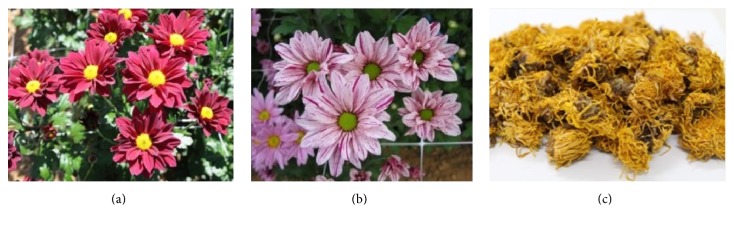
The flowers of (a) a radiation-induced mutant cultivar,* C. morifolium* cv. ARTI-Dark Chocolate (ADC); (b) the original cultivar,* C. morifolium* cv. Noble Wine (NW); (c) the commercially available medicinal herb,* C*.* morifolium *(CM).

Numerous varieties and cultivars have been developed by hybridization and mutation for horticultural purposes [6] and to improve crop productivity and quality [7]. Mutation is widely used in plant breeding research and generated by spontaneous mutation, ultraviolet lights, chemical mutagens, and ionizing radiation (i.e., X-rays and gamma-rays) [7]. More than 3,000 mutant varieties of plants have been registered with the Food and Agriculture Organization/International Atomic Energy Agency (FAO/IAEA), most of which were developed with gamma-irradiation (available online at http://mvd.iaea.org). Recently, our research group has developed a new chrysanthemum cultivar*, C. morifolium* cv. ARTI-Dark Chocolate (ADC), by gamma-irradiation on stem cuttings of the chrysanthemum cultivar,* C. morifolium* cv. Noble Wine (NW), and registered a new plant variety (registration number 4996) in the Korea Seed and Variety Service (available online at https://www.seed.go.kr/english/function/system_06.jsp) (Figure 1).

As part of our ongoing search for functional resources from new cultivars developed by radiation-induced mutation breeding, we assessed a 95% ethanol extract of the flowers of ADC. The extract showed higher antioxidant activity in the DPPH and ABTS radical scavenging assays than did extracts of the original cultivar, NW, and the commercially available medicinal herb* C*.* morifolium* with yellow petals (CM). Therefore, the ethanol extract was subjected to bioactivity-guided fractionation, leading to the isolation of seven flavonoids. In addition to the flavonoids isolated, four phenolic acids were found to be the constituents of the flowers of ADC by HPLC-DAD-ESIMS analysis. To identify the differences in the phytochemical contents of the three flowers depending on their activities, HPLC-DAD-ESIMS analysis was performed on standards and samples of the 95% ethanol extracts of each of the flowers.

## 2. Materials and Methods

### 2.1. General

The 1D NMR experiment was performed on a JNM-ECA 500 MHz NMR instrument (JEOL, Ltd., Tokyo, Japan). Thin-layer chromatographic (TLC) analysis was performed on Kieselgel 60 F254 (Merck, Darmstadt, Germany), with visualization performed under UV light (254 and 365 nm) and 10% (v/v) sulfuric acid spray followed by heating (200°C, 2 min). Silica gel (230–400 mesh, Merck), YMC Gel ODS-A (12 nm, S-150 *μ*m; YMC Co., Kyoto, Japan), and Sephadex LH-20 (Pharmacia Co., Uppsala, Sweden) were used for column chromatography (CC). Analytical high-performance liquid chromatography-diode array detector-electrospray ionization mass spectrometry (HPLC-DAD-ESIMS) was carried out on an Agilent 1200 series system and an Agilent 6120 quadrupole MS system (Agilent Technologies Co., Santa Clara, CA, USA) equipped with a YMC-Triart C18 column (5 *μ*m, 250 mm × 4.6 mm, YMC Co.). Preparative HPLC was performed on a Gilson Preparative HPLC system (Gilson Inc., Middleton, WI, USA) equipped with YMC Pack Pro C18 (5 *μ*m, 250 mm × 20 mm, YMC Co.). A [^60^Co]  *γ*-irradiator (150 TBq capacity; ACEL, Ontario, Canada) was used for gamma-irradiation. The standard compounds chlorogenic acid, 1,4- dicaffeoylquinic acid (DCQA), and 4,5-DCQA were purchased from Wuhan ChemFaces Biochemical Co., Ltd. (Hubei, China). 1,3-, 1,5-, 3,4-, and 3,5-DCQAs were purchased from Chengdu Biopurify Phytochemicals Ltd. (Chengdu, China). 2,2-Diphenyl-1-picrylhydrazyl (DPPH), 2,20-azino-bis(3-ethylbenzthiazoline-6-sulphonic acid) (ABTS), and ascorbic acid were obtained from Sigma Chemical Co. (St. Louis, MO, USA). All other chemicals and solvents used in this study were of analytical grade.

### 2.2. Plant Material

*C. morifolium* cv. ARTI-Dark Chocolate was produced in the same manner described by Jo et al. [8].* C. morifolium* cv. ARTI-Dark Chocolate with dark purple petals was developed by 50 Gy gamma-irradiation from a labeled Cobalt (^60^Co) source on stem cuttings of* C. morifolium* cv. Noble Wine, which is a spray-type chrysanthemum cultivar that has white petals with purple stripes. And then, ADC flowers were selected according to petal-color variants and were examined to be of stable inheritance of phenotype for four years (2009–2012). ADC has been grown by Drs. Y. D. Jo, S. H. Kim, J.-B. Kim, and S.-Y. Kang and has been registered as a new plant variety in the Korea Seed and Variety Service (April 2014). ADC and NW flowers were handpicked and randomly collected at the stage of fully open-flowering with open dick flowers in the same plantation, in October 2015. The flowers were then freeze-dried, ground, and finally stored at −20°C in polyethylene plastic bags until further analysis. Dried flowers of* C. morifolium* with yellow petals, which are used for a medicinal herb, were purchased from the Jewondang herb shop in Jeongeup-si, Jeollabuk-do, Korea. The voucher specimens have been deposited at the Advanced Radiation Technology Institute, Korea Atomic Energy Research Institute.

### 2.3. Extraction and Isolation

The dried flowers of ADC (2 kg) were extracted with 95% EtOH (3 × 30 L) overnight at room temperature. The solvent was evaporated in vacuo to afford a 95% EtOH extract (200 g), which was then suspended in distilled water (2 L) and partitioned with* n*-hexane (3 × 2 L), CHCl_3_ (3 × 2 L), EtOAc (3 × 2 L), and* n*-BuOH (3 × 2 L), sequentially. The EtOAc-soluble fraction (8 g) was subjected to silica gel column chromatography (CC) (CHCl_3_–MeOH, 10 : 0 to 1 : 9, v/v) to yield fifteen fractions (F01–F15). Fraction F06 (200 mg), eluted with CHCl_3_–MeOH (98 : 2) from the first separation, was subjected to Sephadex LH-20 CC (100% MeOH) to give four subfractions (F0601–F0604). Subfraction F0604 (19.5 mg) was separated by preparative HPLC (YMC-Triart C18, CH_3_CN-water, 1 : 1, v/v, 4 mL/min, UV 280 nm) to afford** 1** (*t*_R_ 20.5 min, 3.5 mg) and** 2** (*t*_R_ 40.2 min, 5.3 mg). Fraction F08 (500 mg) was subjected to silica gel CC (CHCl_3_–MeOH, 95 : 5 to 9 : 1, v/v) to yield eight fractions (F0801–F0808). Subfraction F0807 (32.5 mg) was chromatographed on a Sephadex LH-20 (100% MeOH), providing** 3** (25.0 mg). Fraction F10 (994 mg) was subjected to silica gel CC (CHCl_3_–MeOH, 9 : 1 to 1 : 9, v/v) to yield** 4** (29.0 mg). Fraction F13 (800 mg) was applied to RP-C_18_ CC (MeOH–CH_3_CN–water, 1 : 1 : 2 to 1 : 1 : 0, v/v), affording** 7** (2.0 mg). Subfraction F1304 (15.5 mg) was purified by preparative HPLC (YMC-Triart C18, CH_3_CN-water, 1 : 1, v/v, 4 mL/min, UV 280 nm) to yield** 5** (*t*_R_ 20.5 min, 5.5 mg). Fraction F14 (1.18 g) was subjected to RP-C_18_ CC (MeOH–water, 1 : 1 to 2 : 1, v/v), affording** 6** (38.7 mg).

### 2.4. Preparation of Sample and Standard Solutions

Dried flowers of ADC (30 g), NW (30 g), and CM (30 g) were extracted three times with 95% EtOH (200 mL) for 24 h at room temperature, respectively. The extracted solutions were filtered through filter paper and evaporated in vacuo to afford dryness to each (ADC, 6.27 g, w/w 20.9%; NW, 7.96 g, w/w 26.53%; CM, 9.93 g, w/w 33.1%). All 95% EtOH extracts were weighed accurately and dissolved in MeOH at 10 mg/mL. The sample solution was filtered through a syringe filter (0.45 *μ*m) for HPLC analysis. The standards were weighed accurately and dissolved in MeOH at 1.0 mg/mL. The stock solutions were diluted to yield a series of standard solutions at four different concentrations (25, 50, 75, and 100 *μ*g/mL) for quantitative analysis.

### 2.5. Analysis of Phenolic Compounds

Quantitative analysis was conducted using the Agilent 1200 series LC system coupled online with an Agilent 6120 quadrupole single mass spectrometer detector. Data acquisition and processing were performed using the ChemStation software, with an YMC-Triart C18 column (5 *μ*m, 250 mm × 4.6 mm, YMC Co.). Binary gradient elution with 0.1% formic acid in water (v/v, solvent A) and 0.1% formic acid in acetonitrile (v/v, solvent B) was performed as follows: 0–60 min, 15–35% B; 60–70 min, 35–60% B; 70-71 min, 60–95% B; 71–80 min, 95% B; 80-81 min, 95–15% B; 81–90 min, 15% B. The total flow rate was maintained at 0.8 ml/min and the injection volume was 10 *μ*L. Chromatograms were acquired at 265, 280, 330, and 360 nm by the DAD detector. Mass spectra were measured between* m/z* 100 and 1000 in positive ionization mode (ESI^+^) at a scan rate of 1.06 sec/cycle and was monitored by a diode array detector. The mass spectrometric conditions were as follows: capillary voltage = 4000 V; drying gas flow = 10 L/min (N_2_); nebulizer pressure = 30 psig; drying gas temperature = 350°C.

### 2.6. Evaluation of Antioxidant Activity

Antioxidant activities of the flowers were measured using the following assays.

#### 2.6.1. DPPH Free Radical Scavenging Activity

The DPPH of the 95% EtOH extract of each plant materials was determined by Brand-Williams's method [9]. Briefly, each extract was suspended in DMSO and 100 *μ*L of the sample was reacted with 100 *μ*L of 0.2 mM DPPH solution. Absorbance measurements were taken 30 min after the reaction at 517 nm using an ELISA reader (Benchmark Plus, Bio-Rad, Hercules, CA, USA). The concentration of the extract was calculated from the log-dose inhibition curve for 50% inhibition of free radicals (SC_50_).

#### 2.6.2. ABTS Radical Cation Scavenging Activity

The ABTS of the 95% EtOH extract of each type of plant and the solvent fractions of ADC was evaluated using the method published by Re et al. [10]. In brief, the ABTS was measured by preformed radical monocation. The mixtures, along with 7.4 mM ABTS solution and 2.6 mM potassium persulfate, were incubated at room temperature in the dark for 24 hours. The ABTS solution was diluted with phosphate-buffered saline (pH 7.4) to achieve an absorbance of 0.7 ± 0.03 at 732 nm. Each of the samples was suspended in DMSO and 50 *μ*L of the sample was reacted with 950 *μ*L of the ABTS solution. Absorbance was taken 10 min after the reaction at 732 nm using an ELISA reader (Benchmark Plus, Bio-Rad, Hercules). The concentration of the extract was calculated from the log-dose inhibition curve for 50% inhibition of free radicals (SC_50_).

### 2.7. Statistical Analysis

Each experiment was done in triplicate and all data are presented as the mean ± standard deviation (SD). Statistical differences were determined using Student's *t*-test. The significant level was set at *p* < 0.05.

## 3. Results and Discussion

The 95% ethanol extract of ADC, NW, and CM were evaluated for their antioxidant activity using the ATBS and DPPH radical scavenging assays. As shown in Table 1, ADC extract showed higher radical scavenging activity than did NW and CM extract. Therefore, ADC extract was sequentially partitioned with* n*-hexane, chloroform, ethyl acetate, and* n*-butanol and the solvent fractions were also tested for their ABTS radical scavenging activities (Table 1). Among those tested, the ethyl acetate-soluble fraction of ADC exhibited potent inhibitory activity, with an SC_50_ value of 42.84 *μ*g/mL, and it was further subjected to bioactivity-guided fractionation for the isolation of active lead compounds. The antioxidant activity of ADC could presumably be attributed to certain antioxidant components, for instance, phenolic compounds, which has a wide range of health benefits [11].

**Figure 2:**
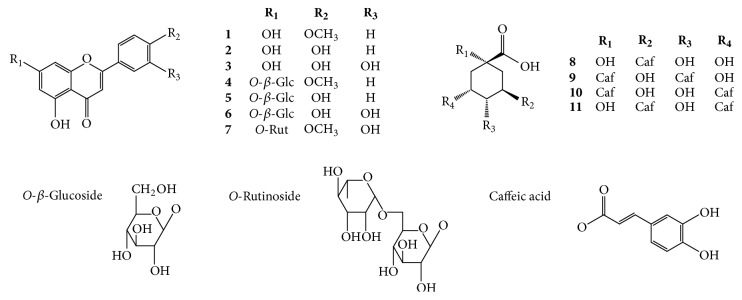
Chemical structures of compounds found in ADC flowers.

**Figure 3:**
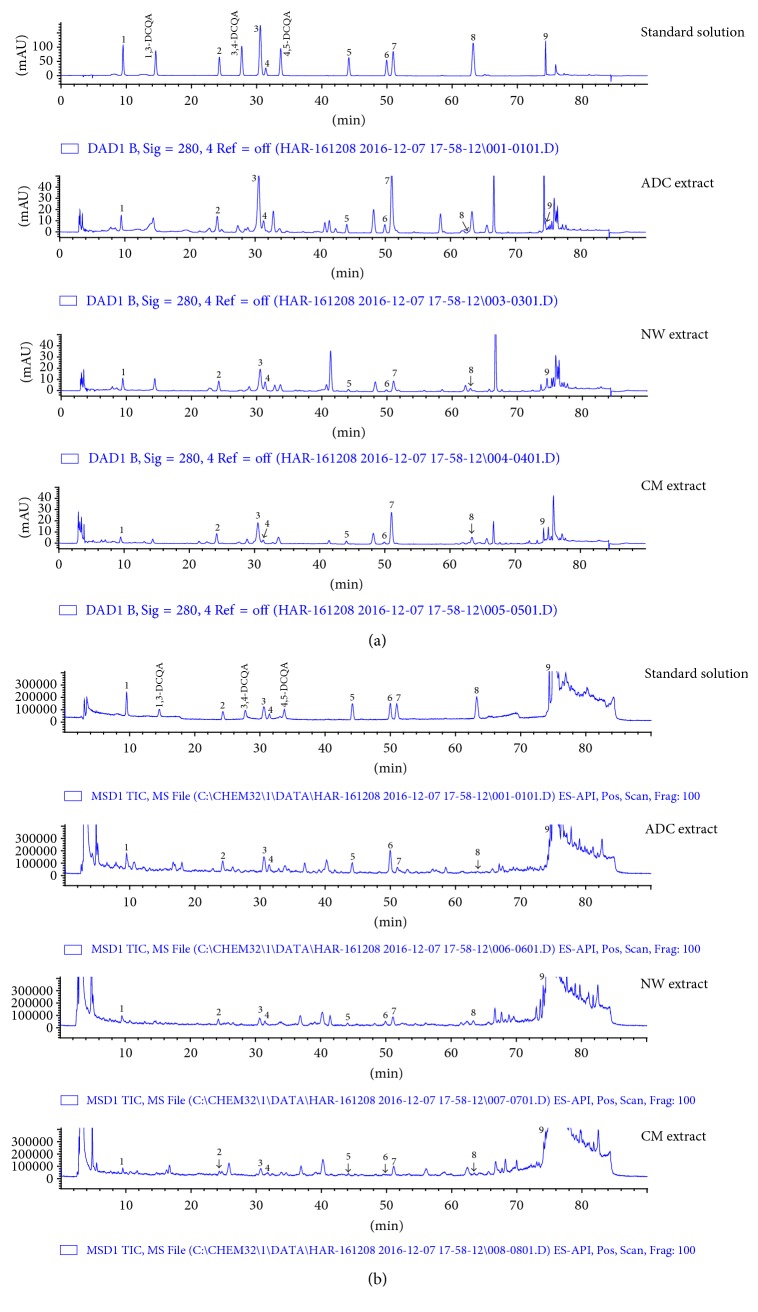
(a) HPLC chromatograms at 280 nm and (b) the expansion of total ion chromatograms in the positive ion mode. For the identification of each numbered peak, see Table 2.

To find out the different phytochemical profiles among ADC, NW, and CM depending on their activities, HPLC-DAD-ESIMS analysis was performed on standards and samples of each 95% ethanol extract of the three flowers. Seven compounds were isolated from the ethyl acetate fraction of ADC and were identified as acacetin (**1**) [12], apigenin (**2**) [13], luteolin (**3**) [14], acacetin-7-*O*-*β*-glucoside (**4**) [13], apigenin-7-*O*-*β*-glucoside (**5**) [14], luteolin-7-*O*-*β*-glucoside (**6**) [14], and linarin (acacetin-7-*O*-rutinoside,** 7**) [15], by analysis of their spectroscopic data as well as by comparison of their data with published values (Figure 2). Peaks for these compounds in HPLC-DAD spectrum were comprehensively determined by comparing their retention times and by coinjection of each sample with standards. (Figure 3; see Figure S1 in Supplementary Material available online at https://doi.org/10.1155/2017/1254721). Peaks 1 and 3 were assigned to chlorogenic acid and dicaffeoylquinic acids by ESI-MS analysis and by comparison with the authentic standards. Their mass ions were readily observed using total ion chromatography (Figures 3 and S2). Peak 1 (*t*_R_ 9.5) gave a molecular ion peak at* m/z* 355.1 [M+H]^+^ and the fragmental mass ion of caffeic acid at* m/z* 163.0 [M-quinic acid]^+^ in its mass spectrum, corresponding to chlorogenic acid (**8**). Peak 3 (*t*_R_ 30.6) demonstrated a molecular ion peak at* m/z* 517.1 [M+H]^+^ and fragmental mass ions at* m/z* 499.1 [M-H_2_O]^+^ and 163.1 [M-caffeoylquinic acid]^+^, indicating dicaffeoylquinic acids (DCQA). There are six positional isomers of DCQAs, such as 1,3-, 1,4-, 1,5-, 3,4-, 3,5-, and 4,5-DCQAs [16], and they are available from commercial suppliers. By comparing their retention times with those of authentic standards, peak at *t*_R_ 30.6 was identified as a mixture of three compounds, 1,4-DCQA (**9**), 1,5-DCQA (**10**), and 3,5-DCQA (**11**), which showed the same retention time in the chromatograms. Other standards, 1,3-, 3,4-, and 4,5-DCQAs, were not detected by total ion chromatography. As a result, nine peaks (peaks 1–9) have been detected, of which the identities and properties are summarized in Table 2.

A quantitative analysis of flavonoids and phenolic acids found in ADC was performed using HPLC-DAD-ESIMS. The established method is described in Section 2.5. HPLC-DAD-ESIMS chromatograms of the 95% ethanol extract of the three flowers and the standard solution are shown in Figure 3 and data for each peak are listed in Table 2. The linear relationships between the peak areas (*y*) and concentrations (*x*, *μ*g/ml) of the compounds were calculated by regression equations (*y* = *ax* + *b*; *a*: slope; *b*: intercept). The calibration curves showed a high degree of linearity with a correlation coefficient of* r*^2^ > 0.999 over the concentration range 25–100 *μ*g/ml. The limits of detection (LOD) and limits of quantification (LOQ) for the 8 compounds and a mixture of** 9**–**11** were in the range of 0.057–0.716 *μ*g/ml and 0.173–2.168 *μ*g/ml, respectively. This analytical method was applied to the simultaneous quantification of the 8 compounds and a mixture of compounds** 9**–**11** from the three flowers, and the results are shown in Table 3. An increase in flavonoid glycosides and phenolic acids and a decrease in flavone aglycones were observed in ADC extract, compared to the extract from NW and CM (Table 4), indicating that radiation-induced mutation breeding enhanced the production of certain active compounds. For examples, apigenin (**2**) [17], luteolin (**3**) [18], luteolin-7-*O*-*β*-glucoside (**6**) [18], chlorogenic acid (**8**) [19], and 1,5-DCQA (**10**) [20] have exhibited their antioxidant effects by attenuating the oxidative damage thorough activation of nuclear factor-erythroid 2-related factor 2 (Nrf2) pathway. There have also been reports that acacetin-7-*O*-*β*-glucoside (**4**) showed moderate DPPH radical scavenging activity [21]; however 3,5-DCQA (**11**) exhibited strong DPPH and ABTS radical scavenging and ferric reducing antioxidant power (FRAP) activities [22]. According to these previous reports, the high content of total phenolic compound in ADC extract was most likely responsible for its antioxidant activity.

Studies on different types of flavonoids and their known functions inside the plant have reported that flavonoid glycosides were involved in protection against radiation stress and in producing purple pigment [23]. In addition, there have been reports that the generation of caffeoylquinic acids was induced in an anthocyanin-accumulating plant cell line such as a purple sweet potato [24], and eventually the biosynthetic pathway of phenolic compounds can be closely related to that of anthocyanins [25]. Thus, a color change to purple of ADC flower petal was considerably affected by radiation-induced mutagenesis and seems to be mediated by the accumulation of flavonoid glycosides and caffeoylquinic acids.

## 4. Conclusion

In conclusion, the radiation-induced mutant cultivar, ADC extract, exhibited stronger antioxidant activity, compared to the extracts from the control plant, NW, and the medicinal herb, CM. The enhancement of the antioxidant properties of ADC flower extract is due to higher concentrations of antioxidant phenolic compounds. Phenolic profiles determined by HPLC-DAD-ESIMS revealed seven flavonoids and four phenolic acids which were tentatively identified and quantified. Therefore, these results make ADC flowers a very promising source of natural-occurring antioxidants as well as a commercially useful product. Further studies on its processing suitability analysis and detailed mechanism of action in our laboratory are being developed for commercialization of ADC flower as a new purple colored chrysanthemum tea or a dietary supplement.

## Supplementary Material

Figure S1: LC-DAD-ESIMS (in positive ionization mode) chromatograms of (A) the mixed compounds solution; (B) 95% ethanol extract of the flowers of ADC; (C) NW; (D) CM. Peaks 1: chlorogenic acid; 2: luteolin-7-*O*-*β*-glucoside; 3: mixture of 1,4-, 1,5-, and 3,5-DCQAs; 4: apigenin-7-*O*-*β*-glucoside; 5: linarin; 6: acacetin-7-*O*-*β*-glucoside; 7: luteolin; 8: apigenin; 9: acacetin.
Figure S2: Mass spectra analysis for peaks 1-9 extracted from total ion chromatography of ADC. Peaks 1: chlorogenic acid; 2: luteolin-7-*O*-*β*-glucoside; 3: mixture of 1,4-, 1,5-, and 3,5-DCQAs; 4: apigenin-7-*O*-*β*-glucoside; 5: linarin; 6: acacetin-7-*O*-*β*-glucoside; 7: luteolin; 8: apigenin; 9: acacetin.

## Figures and Tables

**Table 1:** Antioxidant activities of the extracts and fractions.

Sample	ABTS (SC_50_, *μ*g/ml)	DPPH (SC_50_, *μ*g/ml)
95% ethanol extract of CM	573.74 ± 36.16	1904.20 ± 36.71
95% ethanol extract of NW	1156.00 ± 78.10	1844.82 ± 72.38
95% ethanol extract of ADC	272.32 ± 14.76	960.24 ± 39.63
Hexane fraction of ADC	1464.89 ± 39.90	NT
Chloroform fraction of ADC	282.89 ± 11.00	NT
Ethyl acetate fraction of ADC	42.84 ± 1.10	NT
*n*-Butanol fraction of ADC	269.88 ± 5.75	NT
Aqueous fraction of ADC	1489.12 ± 44.74	NT
Ascorbic acid	16.68 ± 1.05	4.67 ± 0.12

Values (mean ± SD) of extracts and fractions analyzed individually in triplicate; ascorbic acid was used as a positive control; NT: not tested.

**Table 2:** Identification and mass spectrometric properties of the phenolic compounds from chrysanthemum cultivars.

Peak	*t*_R_ (min)	*λ*_max_ (nm)	[M]^+^ (*m*/*z*)	MS^n^ (*m*/*z*)	Compound
1	9.51	220, 325	355.1	163.0	Chlorogenic acid (**8**)
2	24.27	255, 270, 350	449.1	—	Luteolin-7-*O*-*β*-glucoside (**6**)
3	30.61	220, 325	517.1	499.1/163.1	Mixture of 1,4-, 1,5-, and 3,5-DCQAs (**9**–**11**)
4	31.39	265, 340	433.1	—	Apigenin-7-*O*-*β*-glucoside (**5**)
5	44.18	265, 330	593.2	—	Linarin (**7**)
6	49.98	265, 330	447.1	—	Acacetin-7-*O*-*β*-glucoside (**4**)
7	51.08	265, 350	287.0	—	Luteolin (**3**)
8	63.41	250, 260, 350	271.1	223.1	Apigenin (**2**)
9	74.42	265, 335	285.1	—	Acacetin (**1**)

**Table 3:** Linear range, regression equation, correlation coefficients, LODs, and LOQs of the compounds.

Compound	Regression equation	Correlation coefficient (*r*^2^)	LOD (*μ*g/mL)	LOQ (*μ*g/mL)
Acacetin (**1**)	*y* = 11.349*x* + 2.520	0.9996	0.057	0.173
Apigenin (**2**)	*y* = 36.953*x* − 46.980	0.9992	0.575	1.742
Luteolin (**3**)	*y* = 23.094*x* + 5.220	0.9995	0.244	0.738
Acacetin-7-*O*-*β*-glucoside (**4**)	*y* = 14.277*x* + 20.120	0.9991	0.206	0.624
Apigenin-7-*O*-*β*-glucoside (**5**)	*y* = 33.317*x* − 9.170	0.9992	0.542	1.644
Luteolin-7-*O*-*β*-glucoside (**6**)	*y* =14.105*x* − 16.180	0.9993	0.267	0.811
Linarin (**7**)	*y* = 21.849*x* − 21.340	0.9994	0.716	2.168
Chlorogenic acid (**8**)	*y* = 18.675*x* − 17.907	0.9994	0.178	0.539
Mixture of 1,4-, 1,5-, and 3,5-DCQAs (**9**–**11**)	*y* = 22.766*x* − 25.32	0.9991	0.601	1.822

*y* = peak area, *x* = concentration (*μ*g/ml), *a* = slope, *b* = intercept; limit of detection (LOD): 3.3 × (SD of the response/slope of the calibration curve); limit of quantification (LOQ): 10 × (SD of the response/slope of the calibration curve).

**Table 4:** Contents of compounds **1**–**8** and a mixture of **9**–**11** in the three flowers (Mean ± SD, *n* = 3).

Compound	Contents (mg/g)		
	ADC	NW	CM
Acacetin (**1**)	0.529 ± 0.034	0.381 ± 0.019	0.455 ± 0.019
Apigenin (**2**)	0.114 ± 0.004	0.290 ± 0.012	0.251 ± 0.018
Luteolin (**3**)	0.348 ± 0.012	0.577 ± 0.022	1.171 ± 0.066
Acacetin-7-*O*-*β*-glucoside (**4**)	2.441 ± 0.079	0.328 ± 0.012	0.110 ± 0.002
Apigenin-7-*O*-*β*-glucoside (**5**)	0.479 ± 0.010	0.205 ± 0.004	0.076 ± 0.005
Luteolin-7-*O*-*β*-glucoside (**6**)	2.761 ± 0.032	0.629 ± 0.010	0.888 ± 0.103
Linarin (**7**)	0.582 ± 0.010	0.117 ± 0.003	0.211 ± 0.002
Chlorogenic acid (**8**)	0.905 ± 0.010	0.373 ± 0.017	0.533 ± 0.029
Mixture of 1,4-, 1,5-, and 3,5-DCQAs (**9**–**11**)	4.403 ± 0.082	0.833 ± 0.028	1.830 ± 0.077
Total phenolic compounds	12.562 ± 0.255	3.732 ± 0.108	5.525 ± 0.115
