# Supplementary material for: Quantification of Antioxidant Phenolic Compounds in a New Chrysanthemum Cultivar by High-Performance Liquid Chromatography with Diode Array Detection and Electrospray Ionization Mass Spectrometry
Source: Int J Anal Chem. 2017 May 24;2017:1254721. doi: 10.1155/2017/1254721 (PMC5463162; doi:10.1155/2017/1254721)
Supplement: Supplementary file 1 — Figure S1: LC-DAD-ESIMS (in positive ionization mode) chromatograms of (A) the mixed compounds solution; (B) 95% ethanol extract of the flowers of ADC; (C) NW; (D) CM. Peaks 1: chlorogenic acid; 2: luteolin-7-O-β-glucoside; 3: mixture of 1,4-, 1,5-, and 3,5-DCQAs; 4: apigenin-7-O-β-glucoside; 5: linarin; 6: acacetin-7-O-β-glucoside; 7: luteolin; 8: apigenin; 9: acacetin. Figure S2: Mass spectra analysis for peaks 1-9 extracted from total ion chromatography of ADC. Peaks 1: chlorogenic acid; 2: luteolin-7-O-β-glucoside; 3: mixture of 1,4-, 1,5-, and 3,5-DCQAs; 4: apigenin-7-O-β-glucoside; 5: linarin; 6: acacetin-7-O-β-glucoside; 7: luteolin; 8: apigenin; 9: acacetin. [file 1254721.f1.doc]

Supplementary Materials

Quantification of Antioxidant Phenolic Compounds in a New Chrysanthemum Cultivar by High-performance Liquid Chromatography with Diode Array Detection and Electrospray Ionization Mass Spectrometry

**Ah-Reum Han,^1^ Hyo Young Kim,^1^ Yangkang So,^1^ Bomi Nam,^1,2^ Ik-Soo Lee,^2^ Joo-Won Nam,^3^ Yeong Deuk Jo,^1^ Sang Hoon Kim,^1^ Jin-Baek Kim,^1^ Si-Yong Kang,^1^ and Chang Hyun Jin^1^**

^1^Advanced Radiation Technology Institute, Korea Atomic Energy Research Institute, Jeongeup-si, Jeollabuk-do 56212, Republic of Korea

^2^College of Pharmacy, Chonnam National University, Gwangju 11686, Republic of Korea

^3^College of Pharmacy, Yeungnam University, Gyeongsan-si, Gyeongsangbuk-do 38541, Republic of Korea

Correspondence should be addressed to Ah-Reum Han; arhan@kaeri.re.kr

FIGURE S1: LC-DAD-ESIMS (in positive ionization mode) chromatograms of (A) the mixed compounds solution; (B) 95% ethanol extract of the flowers of ADC; (C) NW; (D) CM. Peaks 1: chlorogenic acid; 2: luteolin-7-*O*-*β*-glucoside; 3: mixture of 1,4-, 1,5-, and 3,5-DCQAs; 4: apigenin-7-*O*-*β*-glucoside; 5: linarin; 6: acacetin-7-*O*-*β*-glucoside; 7: luteolin; 8: apigenin; 9: acacetin.


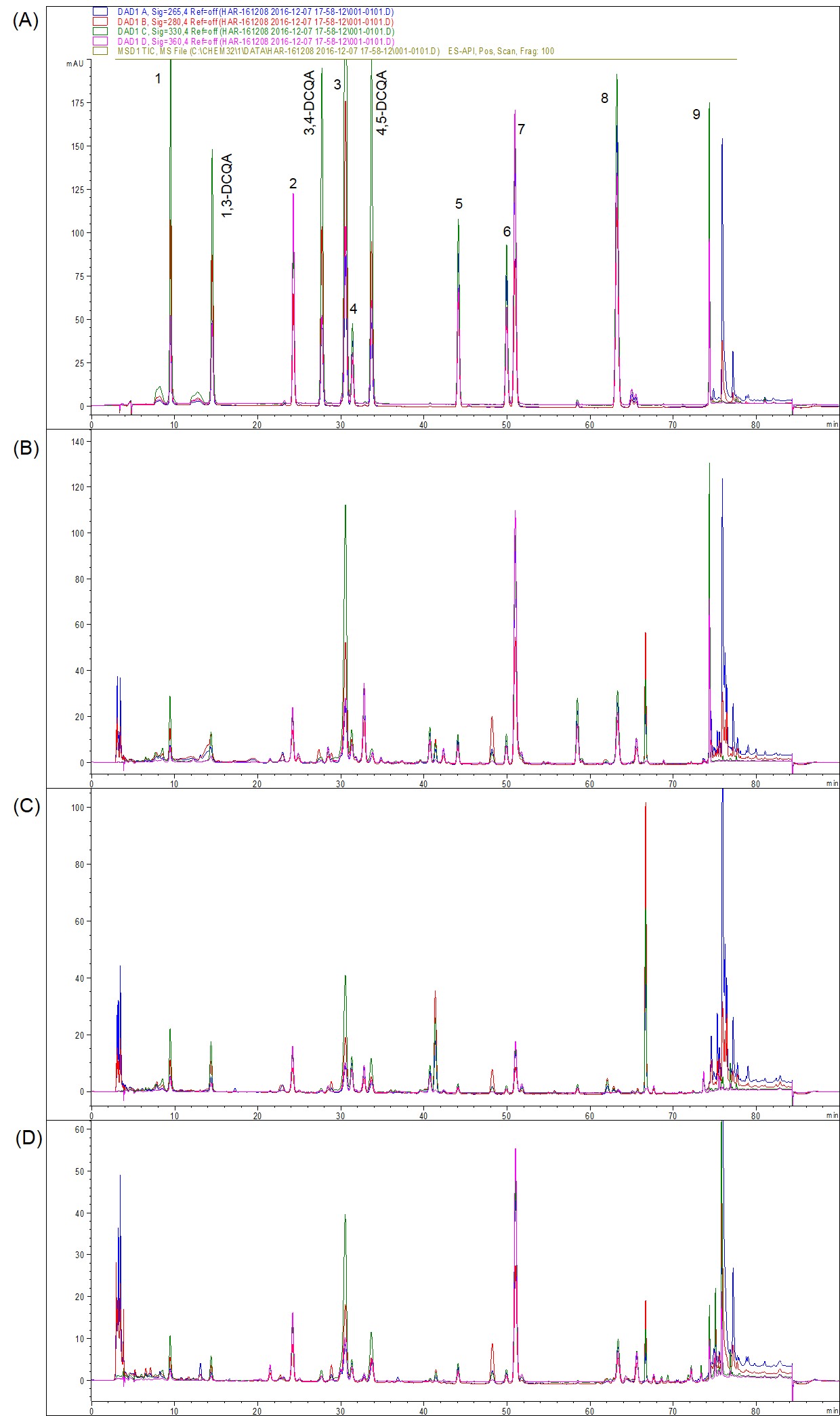


FIGURE S2: Mass spectra analysis for peaks 1-9 extracted from total ion chromatography of ADC. Peaks 1: chlorogenic acid; 2: luteolin-7-*O*-*β*-glucoside; 3: mixture of 1,4-, 1,5-, and 3,5-DCQAs; 4: apigenin-7-*O*-*β*-glucoside; 5: linarin; 6: acacetin-7-*O*-*β*-glucoside; 7: luteolin; 8: apigenin; 9: acacetin.

Peak 1

Peak 2

[M+H]^+^

[M+H]^+^

Peak 3

Peak 4

[M+H]^+^

[M+H]^+^

FIGURE S2: *Cont*.

Peak 5

Peak 6

[M+H]^+^

[M+H]^+^

Peak 8

Peak 7

[M+H]^+^

[M+H]^+^

FIGURE S2: *Cont*.

Peak 9

[M+H]^+^
